# Supplementary material for: Social inequalities in youth mental health in Canada, 2007–2022: a population-based repeated cross-sectional study
Source: Soc Psychiatry Psychiatr Epidemiol. 2025 Jan 17;60(9):2261–6. doi: 10.1007/s00127-025-02813-7 (PMC12378166; doi:10.1007/s00127-025-02813-7)
Supplement: Supplementary file 1 — Supplementary Material 1 [file 127_2025_2813_MOESM1_ESM.docx]

Supplementary Material

Figure S1: Trends in the prevalence of fair/poor self-rated mental health, self-reported mood disorder diagnosis, and self-reported anxiety disorder diagnosis among youth aged 15-24 years, Canadian Community Health Survey 2007-2022


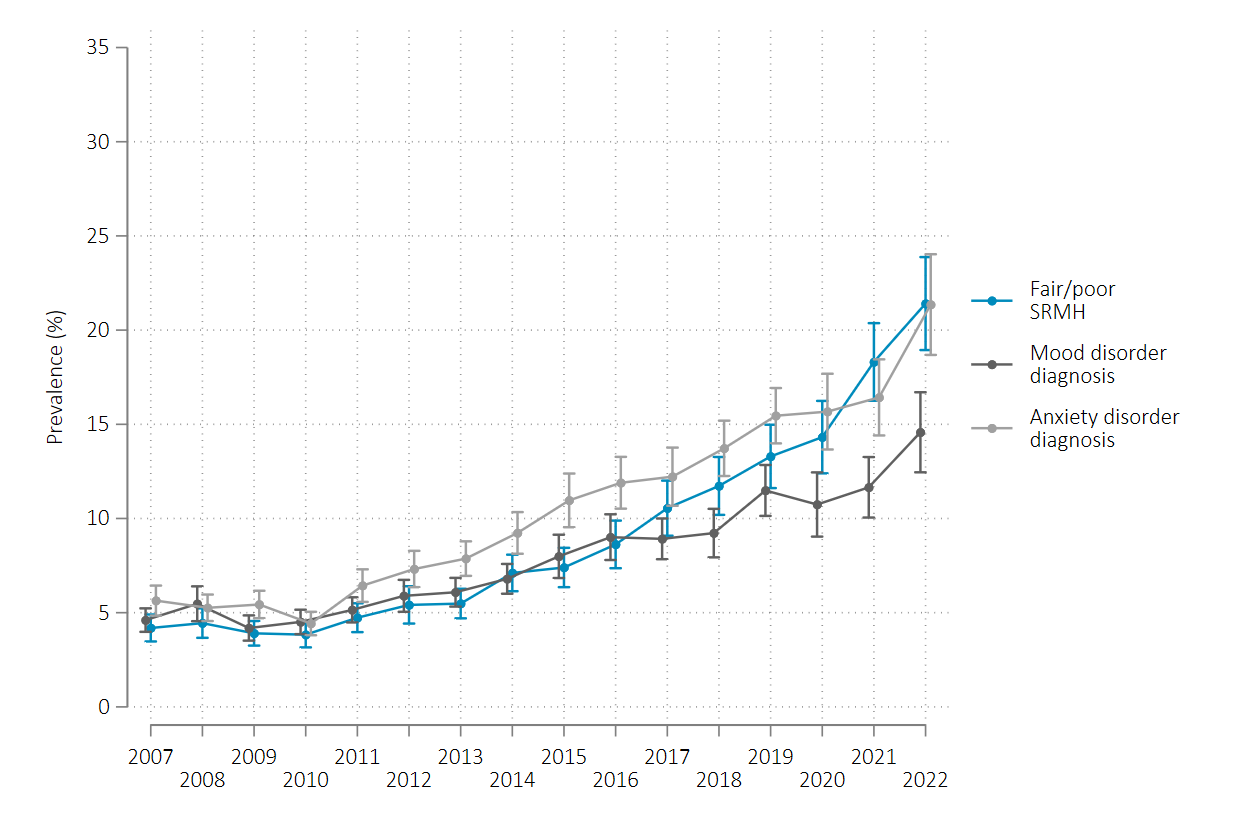


Note: **Self-reported mood disorder diagnosis**: proportion of youth aged 15-24 who reported that they have been diagnosed by a health professional as having a mood disorder, such as depression, bipolar disorder, mania or dysthymia**. Self-reported anxiety disorder diagnosis**: proportion of youth who reported that they have been diagnosed with an anxiety disorder such as a phobia, obsessive-compulsive disorder or a panic disorder.
